# Topological Control of Life and Death in Non-Proliferative Epithelia

**DOI:** 10.1371/journal.pone.0004202

**Published:** 2009-01-15

**Authors:** Camille Martinand-Mari, Benoit Maury, François Rousset, Alain Sahuquet, Gérard Mennessier, Sergei Rochal, Vladimir Lorman, Paul Mangeat, Stephen Baghdiguian

**Affiliations:** 1 Université Montpellier 2, UMR CNRS 5554, Institut des Sciences de l'Evolution, Montpellier, France; 2 Université Montpellier 2, CRBM UMR CNRS 5237, Montpellier, France; 3 Université Montpellier 2, UMR CNRS 5207-LPTA, Montpellier, France; 4 South Federal University, Faculty of Physics, Rostov na Donu, Russia; University of Giessen Lung Center, Germany

## Abstract

Programmed cell death is one of the most fascinating demonstrations of the plasticity of biological systems. It is classically described to act upstream of and govern major developmental patterning processes (*e.g.* inter-digitations in vertebrates, ommatidia in *Drosophila*). We show here the first evidence that massive apoptosis can also be controlled and coordinated by a pre-established pattern of a specific ‘master cell’ population. This new concept is supported by the development and validation of an original model of cell patterning. *Ciona intestinalis* eggs are surrounded by a three-layered follicular organization composed of 60 elongated floating extensions made of as many outer and inner cells, and indirectly spread through an extracellular matrix over 1200 test cells. Experimental and selective ablation of outer and inner cells results in the abrogation of apoptosis in respective remaining neighbouring test cells. In addition incubation of outer/inner follicular cell-depleted eggs with a soluble extract of apoptotic outer/inner cells partially restores apoptosis to apoptotic-defective test cells. The 60 inner follicular cells were thus identified as ‘apoptotic master’ cells which collectively are induction sites for programmed cell death of the underlying test cells. The position of apoptotic master cells is controlled by topological constraints exhibiting a tetrahedral symmetry, and each cell spreads over and can control the destiny of 20 smaller test cells, which leads to optimized apoptosis signalling.

## Introduction

At a branching point between invertebrates and vertebrates [1], *Ciona intestinalis* and other ascidians have emerged as attractive models in the fields of evolution and development. Further interest has recently been raised with the sequencing and annotation of *Ciona intestinalis* and *Ciona savignyi* genomes [2], [3]. The embryonic development of *Ciona* can be experimentally triggered and genetically manipulated [4], with a transparent wild type or mutant juvenile being generated less than 48 h after fertilization. As for other metazoans, apoptosis, in urochordates, is a driving force for developmental processes [5]–[14]. The earliest apoptotic event was observed in follicular cells, a set of epithelial cells that surround the spherical oocyte. Follicular cells of freshly collected, non fertilized eggs turn spontaneously apoptotic (with a completion time of ∼4 h), whereas fertilization delays the time course by a few hours. Interestingly, apoptosis of follicular cells in early embryo was found in phase with hatching, and drug-induced blockade of apoptosis was shown to delay swimming tadpole formation [6].

During on-going studies aimed at deciphering the chronological events leading to the elimination of all follicular cells, we observed that a subset of specialized cells was geometrically pre-positioned through a regular pattern, governed by physical constraints. This subset was shown to exert a spatial and temporal control of the fate of another subset of neighbouring cells. This observation supports the emergence of an original concept according to which biological order (cell patterning) leads to the optimized control of a downstream biological process (an apoptosis cascade).

## Results

### Ciona intestinalis follicular system is composed of 3 types of cells that are subjected to a sequential time-course of apoptosis

**Figure 1:**
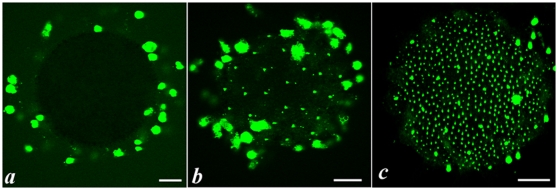
Time course of accessory cell apoptosis in unfertilized eggs of *Ciona intestinalis*. Apoptosis was detected by TUNEL labelling (Roche Molecular Biochemicals kit) and fluorescence microscopy on freshly fixed eggs at the indicated times after collection: a (0 h); b (1 h); c (8 h). Three types of TUNEL positive (green) nuclei of different sizes were characterized during the kinetics. Scale bar: 70 µm.

**Figure 2:**
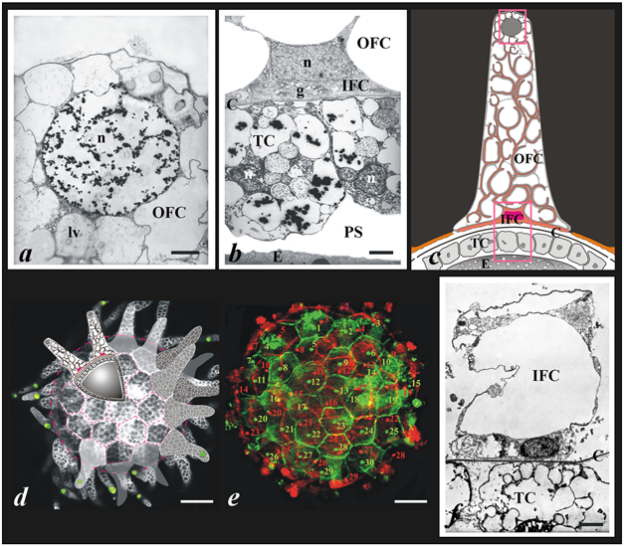
The anatomical organisation of accessory epithelial cells of *Ciona intestinalis* eggs. a–b, f, Ultrastructural characterization of accessory cells of eggs fixed at 0 h (a–b) and 24 h (f) after egg collection. Nuclear chromatin condensation is evidenced only in OFC at 0 h (a), whereas no sign of condensation is present in IFC and TC (b). Note the respective positioning of IFC and TCs across both sides of the chorion. At 24 h, apoptosis was completed in IFC and TC and OFC have disappeared (f). (n; nucleus; g, Golgi apparatus, PS; perivitelline space, E; egg; C, chorion; lv, large vacuole). c, Drawing of an interpretative view of the follicular and test cell organization based on ultrastructural studies (original art by Laurence Meslin and Stephen Baghdiguian, © CNRS-Meslin). Upper and lower insets correspond respectively to the fields shown in a and b. The red-coloured IFC, located at the base of the extension, was characterized as geometrically positioned (see Fig. 1). Indeed, both DAPI staining (not shown) and transmission electron microscopy analysis revealed that such extensions contained only 2 cells, one with a nucleus localized at the bottom of the extension (the IFC), and the second close to the tip of the extension (the OFC). In addition, in the perivitelline space (PS) small TCs form the egg-covering monolayer. Therefore, 3 cell types of very different morphology (OFC, IFC and TC) form *Ciona intestinalis* follicular organization. d is a composite picture of an egg (0 h collection time) labelled with fluorescent phalloidin and TUNEL staining, to which sketches were added to help understanding the general organization of *Ciona* egg. Green apoptotic nuclei of OFCs were localized close to the tip of follicular extensions. It is important to note that if phalloidin labelling (white) allowed a clear discrimination along the boundaries of both TCs and IFCs, a strong F-actin signal was also recorded surrounding the numerous vacuoles present in the floating extensions (original art by Laurence Meslin and Stephen Baghdiguian, © CNRS-Meslin).e is a confocal microscopy reconstruction of *Ciona intestinalis* egg surface, with the two hemispheres being presented in green and red pseudo-colour respectively. 59 IFC (*via* TUNEL positive nuclei) were counted, with 30 green nuclei for one hemisphere and 29 red for the other. This egg is representative of a total of six, from which all TUNEL-positive IFCs were counted in a double blind manner, with care being taken to eliminate signals clearly identified as arising from OFCs (both on the basis of nucleus size and position). Scale bars: a,b and f, 1.5 µm; d and e 40 µm.

During a typical time-course of apoptosis in follicular cells of non fertilized *Ciona intestinalis* eggs, apoptotic nuclei of various sizes were sequentially observed (**Fig. 1**). At egg collection time (**Fig. 1a**), only very large nuclei localized at the tip of follicular extensions were TUNEL-positive. These nuclei disappeared with time whereas a second population, of a smaller size, was then observed (**Fig. 1b**). Interestingly, these nuclei appeared non-randomly positioned (see later). Finally at longer times, a third type of smaller and more numerous nuclei were observed (**Fig 1c**), some of them being already detected at earlier times and located close to the previous ones (**Fig 1b**). Therefore, 3 different cell populations underwent apoptosis with different and sequential time-courses. The 3 cell populations were then characterized at the ultrastructural level (**Fig. 2a–b, f**) and were found homologous to the 3 types of accessory cells previously described in *Ciona savigny* [15], [16]. Based on electron and light fluorescent microscopy observations, a specific model of follicular organization was established for *Ciona intestinalis* (**Fig. 2 c–d**), and was found clearly different from *savignyi*. Additional quantitative analysis supported the following description of *Ciona intestinalis* follicular cell anatomical and topological organization. A monolayer of ∼1200 test cells (TCs) formed a leaky epithelium in the perivitelline space. One membrane domain of TCs faced *Ciona intestinalis* oocyte, whereas the other was closely associated with an extracellular matrix (chorion). On the outer side of the chorion, and tightly associated with it, ∼60 inner follicular cells (IFCs) were each spread over the surface covered by on average 20 TCs (see below and in the legend of **Fig. 2** how cell numbers were determined). IFCs formed the base of an 80 µm-height highly vacuolized finger-like structure, which provided the egg with floating capacity. IFCs were found much larger than TCs and intimately connected with a third type of cells, the outer follicular cells (OFCs), whose largest nucleus was localized at the top of this floating device. This layered epithelial organization correlated well with the sequential order of apoptosis observed in the three cell populations (**Fig. 1** **–** **2**). The number of IFCs was determined through careful complete sectioning of eggs by confocal microscopy and found to be **58.7+/−1.85** (between-egg SE from repeated measure ANOVA). As an example, **Fig. 2e** showed a composite image where both pseudo-coloured hemispheres of the same egg were reconstructed showing all numbered apoptotic nuclei with respect to the IFC contour, which appeared either hexagonal or pentagonal. It should be emphasized that heptagonal, octagonal or more complex contours were never observed. This cellular topology appeared very distinct from what was recently observed in *Drosophila* imaginal disc proliferating epithelium where 20.8% of the cells presented heptagonal shapes [17]. In *Ciona intestinalis*, an average of 20 TCs faced each IFC, the border of the latter being underlined by interrupted red lines in the sketch of **Fig. 2d**.

**Figure 3:**
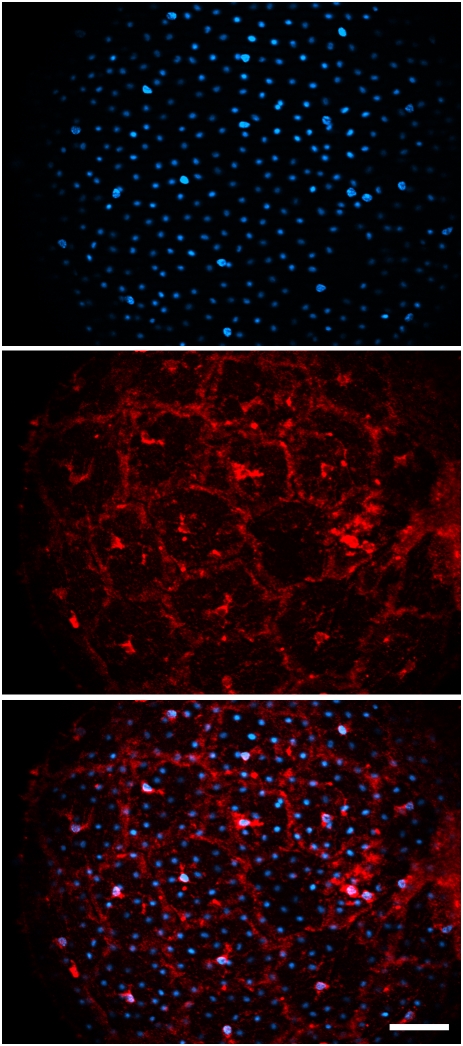
Healthy IFCs express T4. Freshly collected eggs were fixed, permeabilized and immunodetected for T4 (middle panel, red fluorescence). DAPI staining (upper panel, blue fluorescence) allowed the clear cut identification of IFCs (large nuclei) from TCs (small nuclei). Upon merging (lower panel), one can note the distinct localization of T4 immunofluorescence concentrated around the nucleus and the plasma membrane vicinity of IFCs. Scale bar : 30 µm.

During the time-course of the apoptotic events that affected all types of follicular cells, two independent facts were observed. First, OFCs were always found apoptotic as soon as eggs were collected, suggesting that apoptosis was achieved at the end of egg maturation. Second, apoptosis timing in IFCs and TCs was linked, apoptosis being always first observed in IFCs before being triggered in neighbouring TCs. In addition, and in agreement with a previous report [6], apoptosis in both cell types was found to be dependent on fertilization. Indeed fertilization delayed by 4–5 h the appearance of apoptotic nuclei first in IFCs and subsequently in TCs (data not shown). However, fertilization was shown to delay apoptosis in IFC and TC independently of OFC apoptotic status (data not shown). We have already reported that a surge in T4 synthesis induced a transient survival pathway responsible of the observed apoptotic delay [6]. We therefore looked whether T4 synthesis takes place in IFCs (indeed these cells contain secretion granules), because it would be unlikely that we would observe delayed apoptosis in IFC upon fertilization if only TCs synthesize T4. Indeed, in intact freshly collected eggs, T4 appeared to be predominantly synthesized in IFCs (**Fig. 3**). These results support a potential role for IFCs to control and drive apoptotic signalling to neighbouring TCs.

### Inner follicular cells control apoptosis of neighbouring test cells

**Figure 4:**
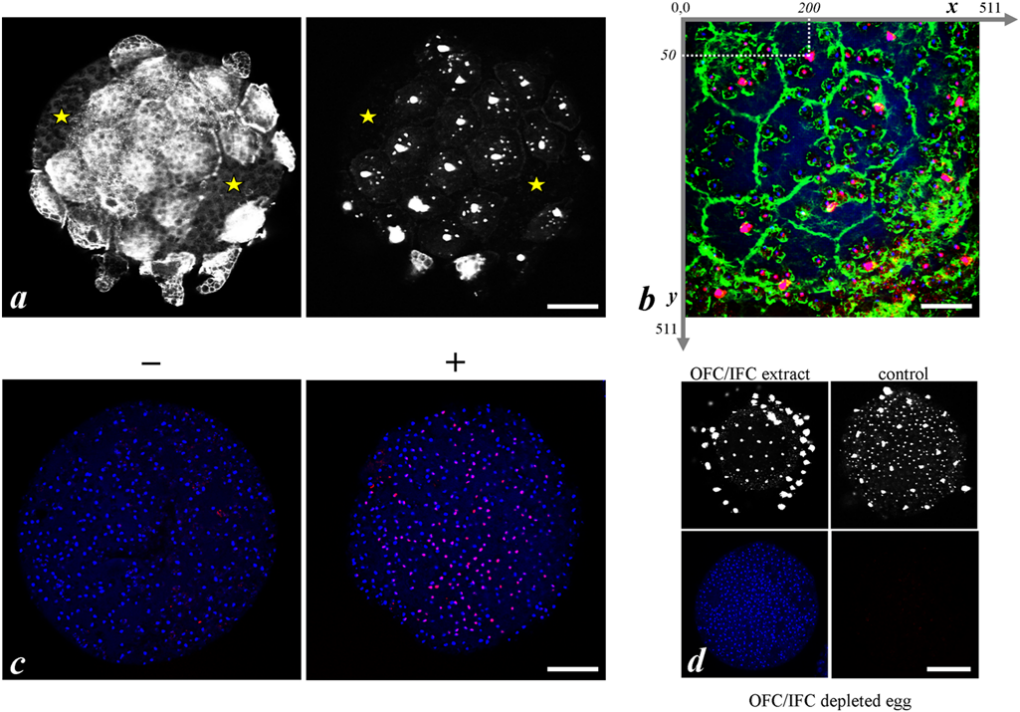
IFCs direct apoptosis in underlying TCs. a presents an example of an egg (4 h collection time) following a mechanical ablation of some floating follicular structures to eliminate OFCs and IFCs, leaving TCs at the cell surface. Phalloidin staining (left panel) validated the residual presence of TCs in ablated areas of the egg where floating extensions were removed and distinguished them (yellow stars) from unaffected ones. TUNEL staining (right panel) of the same field showed that TCs which were no longer linked to IFCs did not become apoptotic. This observation was subjected to a statistical analysis. For that, we reallocated by permutation the *n* observed apoptotic TC nuclei to *n* randomly chosen positions among all observed TC nucleus positions on the surface of intact and mechanically-treated eggs. To determine these positions, intact and mechanically-treated eggs fixed at random times after collection were triple labelled for DAPI, TUNEL and phalloidin staining (b) and observed by confocal microscopy. From each image, the *x*,*y* coordinates of each apoptotic and non apoptotic nuclei were recorded as presented. For each permuted sample, we computed the mean, over all apoptotic TC nuclei, of their distance to the closest IFC nucleus. The distribution of this statistics was estimated from 10,000 permutations and compared to the value of the statistics of the actual sample. Respective P-values computed for each egg were 0.106, 0.0045, 0.0004, 0.3606 and <10^−4^ and when combined by the Z transform method [35] gave a combined P-value of <3. 10^−7^. A similar analysis was performed on 10 eggs from which some IFCs had been ripped off. It should be noted that this experimental situation potentially increases the power of the test because the variance of distance between TC nuclei and the closest IFC nucleus was increased. Individual P-values for each egg were 0.0634, 0.0166, 0.0054, 0.0019, 0.0737, 0.0017, 0.1095, 0.0072, <10^−4^ and <10^−4^, the combined P-value dropping to <4.10^−15^. c Freshly collected non fertilized eggs were immediately depleted of all OFC by a short time mechanical agitation. Depleted eggs were treated (+) or not (−) for 1 h in presence of a soluble extract of apoptotic OFC and IFC (the apoptotic status of the cellular extract was controlled by TUNEL labelling as shown in d/upper left) and finally double labelled with DAPI (blue) and TUNEL(red). d: non fertilized eggs 8 h after collection were mechanically OFC/IFC depleted (lower panels) or not (upper right panel) and labelled with TUNEL (right upper and lower panels) and DAPI (left lower panel). Right and left lower panels showed the same fluorescent field that demonstrated that the remaining TCs in this depleted egg failed to become apoptotic as opposed to control egg (right upper panel). Scale bars : a, 50 µm; b, 20 µm; c, 50 µm; d, 75 µm.

In order to test a potential regulatory role for inner follicular cells in the massive apoptosis observed in the test cell monolayer, we mechanically ablated some floating follicular structures to eliminate the associated OFCs and IFCs, leaving only the respective TCs at the cell egg surface. By comparison with mechanically unaffected areas of the same egg, IFC elimination turned out to prevent TC apoptosis in the selective area (**Fig. 4**). In such a case, the first TC nuclei to become apoptotic should be among the closest from IFC. To verify whether this assumption was right, we performed permutation tests following the standard theory of conditional tests [18]. We compared the observed pattern to random patterns each generated by reallocating the *n* observed apoptotic TC nuclei to *n* randomly chosen positions among all observed TC nucleus positions on the surface of intact or mechanically-treated eggs. For each such permuted sample, we computed the mean, over all positive TC nuclei, of the distance to the closest inner cell nucleus. The distribution of this statistic was estimated from 10,000 permutations and compared to the value of the statistic to the actual sample. This procedure does not make any assumption about the distribution of the test statistic and takes into account that the distances between different pairs of nuclei on the egg surface are not independent from each other. The data of this statistical simulation strongly supported that apoptosis in TCs was directly controlled by the apoptotic status of the neighbouring IFC (see legend of **Fig. 4** for details).

In addition to the mechanical ablation of inner follicular cells approach described above, we developed an assay to verify whether we could induce apoptosis in test cells treated with a soluble extract of inner follicular cells. In order to do so, we first mechanically eliminated all outer and inner follicular cells. In these OFC- and IFC-depleted eggs only test cells remained exposed at egg surface and, as expected, were no longer found apoptotic (**fig 4d** right lower panel) in opposition to control non depleted eggs (**fig 4d** right upper panel). Treatment of OFC/IFC-depleted eggs with a soluble extract made of a mixture of apoptotic outer and inner follicular cells (**fig 4c**) partially induced apoptosis in test cells of 30% eggs. **Fig 4c** showed an example of apoptosis after treatment of eggs with such a soluble extract. This result is consistent with the existence of yet unidentified apoptogenic soluble factor(s) present in inner follicle cells that would diffuse and target underlying test cells through the chorion. For example, 12 kDa-fluorescent dextran was found able to fully cross the chorion barrier and reach the membrane contour of all test cells (data not shown).

### Inner follicular cells are not randomly positioned

It was striking upon observation of TUNEL-positive cells, such as those presented in **Fig 1b–c**, that inner follicular cells were apparently non-randomly distributed over the *Ciona intestinalis* egg surface, and possibly fitting in with an unidentified pattern. In order to test this possibility, we performed permutation tests through which the geometric pattern of TUNEL-positive cells was compared to random geometric patterns each generated by reallocating the positive signals to *n* randomly chosen positions among all observed nucleus positions (*i.e.* “1200”+“60”) on the egg surface. For each of 10,000 such permuted samples, we computed the mean, over all nuclei, of the distance of each positive signal to the closest one. For each of the five eggs analyzed, the P-value was <2.10^−4^, therefore strongly consistent with a regular positioning (pattern) of inner follicular cells over the spherical egg surface.

### Inner follicular cells are geometrically positioned with respect to a tetrahedral symmetry model

**Figure 5:**
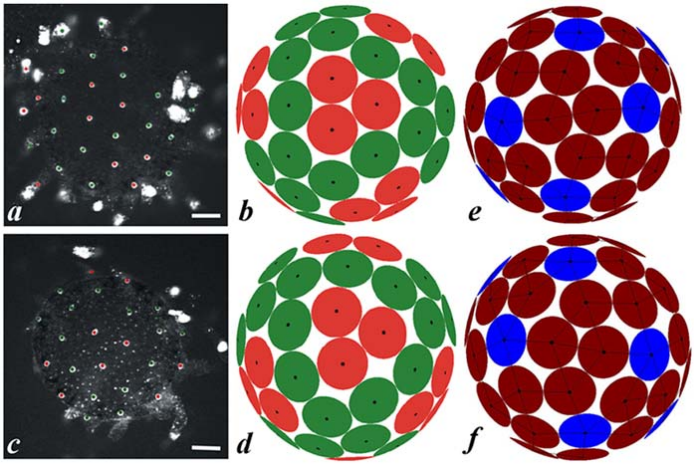
The geometrical order in IFC nucleus position pattern. a, c, Tetrahedral patterns of TUNEL-positive IFC nuclei. Sixty positions form five 12-fold orbits of the symmetry group of all rotations of a regular tetrahedron. b, d, Simulated patterns minimizing interaction energy function (1) for 8<α<12. In Fig. a–d the 3-fold axes of cell distribution are situated in the centres of red triangles and the 2-fold axes are located in the centres of lozenges formed by four coloured-green nuclei. Two different types of 3-fold axes permitted by tetrahedral symmetry with different local IFC nucleus arrangements can be followed in a, b and c, d, respectively. e, f, Best tetrahedral packing configuration. Two hemispheres with 2-fold axes located in the image centres are shown. The positions with five nearest neighbours are coloured in blue, those with six neighbours in brown. The rigidity graph characterizing cell packing in the pattern is obtained by joining the centres of circles which are tangent. 126 edges of rigidity graph are presented by thin solid black lines. Scale bars: a, c, 50 µm.

The next challenge was to develop tools in order to test whether or not physical laws govern the observed pattern of *Ciona intestinalis* inner follicular cells. In non-proliferative epithelia the mutual action of cells resulted in an almost optimal packing. This type of organization led to predictable hexagonal geometrical arrays in the simple case of a flat surface [19]. For an egg, cell patterning is strongly affected by topological constraints imposed by the spherical topology and by the fixed finite number of cells in the IFC monolayer. Similar constraints have been shown to control patterning in simpler biological systems like protein positions in virus capsids [20], [21]. The geometrical order of 60 IFCs positioned on the chorion surface could be characterized either by the regular polygonal pattern of lateral membranes or by the regular set of nucleus positions, both characteristics being intimately related (**Fig. 2e**). Cell nuclei were uniquely described by a surface triangulation graph, in which vertices were associated with nucleus positions, all faces being triangles formed by neighbouring vertices, and edges connecting the nearest neighbours only. Due to the constraints, nucleus positions with the number of nearest neighbours other than 6 should necessarily appear in the pattern. The number of these positions was closely related to the Euler characteristic χ of the triangulated surface. Namely, if in addition to hexavalent positions (those with 6 neighbours) the pattern contained *N_5_* pentavalent positions (with 5 neighbours) and *N_7_* heptavalent ones (with 7 neighbours), the difference *N_5_–N_7_* was equal to 6χ [22]. With χ = 2 for the sphere, the nucleus position pattern of spherical egg should contain at least 12 pentavalent positions (in the case of *N_7_* = 0). Although topology permitted more complicated cell environments, almost all experiments performed on *Ciona intestinalis* eggs have shown patterns with 12 pentavalent cells (**Fig. 2e**). The extensive analysis of nucleus position patterns revealed a (nearly) tetrahedral symmetry of cell distribution (**Fig. 5 a–d**). Sixty observed positions spun five regular 12-fold orbits of the *T* symmetry group (the group of all rotations of a regular tetrahedron).

The emergence of geometrical order in the IFC pattern could be related to two long-standing problems arising in a whole series of biological, physical and chemical systems, namely, the Thomson [23] and Tammes [24] problems. The Thomson problem dealt with the equilibrium positions of *N* unit point charges on the surface of a unit sphere. The aim of Tammes problem was to find the largest diameter of *N* equal circles which could be closely packed on the sphere. In both cases the system of *N* particles had to minimize the interaction energy function of the form:
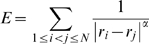
(1)where ***r*** *_i_* were particle positions; with *α* = 1 for Coulombic long-range interaction of charges; circles packing (very short-range interaction) corresponding to *α*→∞. However, neither Thomson nor Tammes problem for N = 60 particles lead to a pattern with tetrahedral symmetry. For *N* = 60 point charges Coulombic interaction led to the distribution with *D_3_* symmetry (rotational symmetry of a triangular prism) [25] while Tammes optimal circle packing resulted in the distribution with trivial *C_1_* symmetry [26]. However, Coulombic potential was of too long-range to model the short ranges interactions classically observed in living cells, whereas Tammes problem was associated with extremely short-range potential but excluding the fluctuations induced by both thermal and active biological intracellular processes.

Epithelial cells interacted by rather short-range hard-core effective potentials of elastic and biochemical origin. Taking nuclei as topological 〈〈point charges〉〉 we modelled their effective interaction energy by function (1) with the value of *α* much greater than 1 but remaining finite. Several modern numerical minimization algorithms [27]–[29] were concurrently used to search the “putative” global minimum of the interaction energy. For *α* ranging from 8 to 12, all algorithms converged to tetrahedral cells configuration. The resulting distribution was quite different from those of both Thomson and Tammes problems but very close to the experimental one (**see** **Fig. 5**). The simulated tetrahedral pattern was given (**Fig. 5 b, d**) with the minimum angular distance between the nuclei of neighbouring cells represented by the angular diameter of circles. Further analysis of the spatial positioning of cells could be done analytically. We could show that apparently, IFCs are preferably organized following a pattern fitting the best packing among different symmetric configurations. For that aim we compared the quantitative characteristics of the best tetrahedral configuration with those of the best octahedral and icosahedral ones of 60 particles studied previously in the frame of physical chemistry problem [30]. The best tetrahedral packing of 60 circles could be obtained analytically by imposing close packing conditions to the tetrahedral distribution obtained numerically. It was characterized by the so-called rigidity graph of the pattern (**Fig. 5 e–f**). The observed minimum angular distance between neighbouring nuclei (*i. e.* the circle diameter divided by the sphere radius, the quantity measured in degrees and independent of the egg size) for the best tetrahedral pattern was *d_T_* = 27.17864°. This value was greater than known for octahedral (*d_O_* = 26.32837°) or icosahedral (*d_I_* = 26.82139°) nearly optimal packings of 60 circles [30]. This analysis confirmed the advantage of the regular tetrahedral pattern of IFCs with respect to all possible symmetric cell configurations in the considered system. Actually, the ordered geometrical organization of IFCs was imposed by topological constraints implying short-range cell-cell interactions. The regular positioning of IFCs played in turn, a decisive role to optimize an apoptotic process in target test cells.

## Discussion

Understanding the laws that control the elaboration of the shape of living organisms is an exciting field of research. Until now, all kinds of organized patterns involving spherical topology were referred to rigid structures (viruses, pollens, fullerenes and so on…). Here, we develop a model of cell patterning applied to a soft spherical object involving short range elastic interactions that fully fits the cellular positioning of inner follicular cells. *Ciona intestinalis* follicular organization exhibits a unique geometrical order characterized by the presence of 60 optimally positioned extensions. IFCs play decisive roles in the generation, maintenance and destiny of the overall structure. Each IFC is orderly positioned with respect to a tetrahedral symmetry. This pattern formation, simulated in an original model of cell positioning, fits with experimental data where 60 cells fully cover the surface of a sphere. Therefore the overall organization of *Ciona intestinalis* follicular structure is dictated by topological constraints.

In addition to its central place in follicular organization, IFCs also play a key role in the massive apoptosis that affects follicular cells, notably test cells. Indeed, ablation experiments **(** **Fig 4c left panel** **)** and assays of re-sensitisation of TC to apoptosis with an OFC/IFC extract **(** **Fig 4c right panel** **)** point to a crucial role for IFC. IFC acts as an ‘apoptotic master cell’, with each IFC exerting a life or death control over 20 neighbouring target TCs. Clearly, both biological assays and statistical analysis demonstrate that the fate of TC is directly controlled by the own fate of IFC. Since an extracellular matrix physically separates both types of cells, it seems likely that some soluble apoptotic signal(s) should be secreted by IFC towards TCs. The exact nature of the signal(s) remains to be established. However it should be noted that T4 can be readily synthesised in healthy IFCs (Fig. 3), those cells therefore are likely to be instrumental in the inducing event that governs the apoptosis delay observed in TCs upon fertilization. To which extent the fate of IFC is governed by OFC is another unanswered question. This is experimentally uneasy to address since OFC were always found apoptotic at egg collection time.

Numerous apoptotic processes leading to organized patterns have been reported in the literature. Here we have described the first case where a pre-established pattern leads to an optimized apoptotic program, by which the death of 60 geometrically-positioned cells controls the death of 1200 more. It is important to point out that the capacity for an IFC to send a death signal toward test cells is not dependent of IFC symmetrical positioning, but that, indeed, IFC pattern makes the apoptotic program particularly fully efficient to target and reach all test cells. This observation, performed in a phylo-genetically pertinent model, is consistent with the statement that biological order (cell patterning) governed by physical (topological) constraints leads to the optimized control of downstream biological process (an apoptosis cascade). To be generalized, this new concept needs to be validated in other model organisms where massive apoptotic events have been previously observed. Indeed, it would be necessary to experimentally re-evaluate in this new perspective some well known cases of programmed cell death such as those occurring for example during cavitation in mammals [31], [32], organopoptosis in *Botryllus* [33], transition of vegetative *Dictyostelium* cells into dead, vacuolated stalk cells, or synchronous developmental cell death observed in *Volvox* [34].

In conclusion, we have provided original observations that entitle us to propose the emergence of two new concepts: on the one hand, the existence of ‘apoptotic master cells’ both susceptible to apoptosis and able to transmit apoptotic signals to other neighbouring cell types, and on the other hand the pre-eminence of topological constraints in the control of life and death within complex epithelial structures.

## Materials and Methods

### Transmission electron microscopy

*Ciona intestinalis* specimens were obtained from Roscoff (France). Eggs, fixed in 2.5% glutaraldehyde in 0.1 M Sörensen phosphate buffer (pH = 7.24) supplemented with 1.5% NaCl, and post-fixed with 2% osmium tetroxide in 0.1 M Sörensen phosphate buffer (pH = 7.4), were dehydrated in graded alcohol series and embedded in Epon 812. Ultra-thin sections, contrasted with uranyl acetate and lead citrate, were observed with a Jeol 1200× transmission electron microscope at 80 kV.

### Immunofluorescence studies

Oocytes, fixed for 20 minutes with 3.7% formaldehyde in filtered seawater at room temperature, were subjected to indirect immunofluorescence (rabbit anti-L-thyroxine (T4) polyclonal antibody from Sigma Immuno Chemicals), Phalloidin-TetramethylRhodamine (from Sigma) and/or TUNEL (kit from Roche Molecular Biochemicals) staining as described previously [5], [6]. Specimens were analyzed with a Leica TCS 4D laser confocal microscope.

### Numerical minimization

The numerical energy minimization algorithm was based on the optimized gradient method. The step value was dependent on previous steps and on gradient variation velocity. Local minima were determined by successive variations of initial conditions.

### Analytical tetrahedral pattern

The analytical form of the tetrahedral nucleus position pattern was obtained by imposing close packing conditions to a tetrahedral distribution obtained numerically. In the tetrahedral structure the positions of nuclei span five 12-fold orbits of the *T* group, their coordinates being weakly dependent on the value of α ranging from 8 to 12. To characterize the tetrahedral pattern analytically it was then sufficient to calculate angular coordinates of five non-equivalent nuclei (with one generating position in each orbit). This was achieved by equalizing the lengths of 11 symmetrically non-equivalent edges of the surface triangulation graph with tetrahedral symmetry. Only three different manners existed to choose these 11 edges, with one corresponding to the best tetrahedral packing of 60 circles. The “rigidity graph” of the pattern was obtained by joining all tangent circles through 126 edges (**fig 3e–f**) passing by the centres and the “kissing points” of tangent circles.
